# An Exploration of the Differential Effects of Parents' Authoritarianism Dimensions on Pre-school Children's Epistemic, Existential, and Relational Needs

**DOI:** 10.3389/fpsyg.2016.02079

**Published:** 2017-01-10

**Authors:** Margherita Guidetti, Luciana Carraro, Luigi Castelli

**Affiliations:** ^1^Dipartimento di Comunicazione ed Economia, University of Modena and Reggio EmiliaReggio Emilia, Italy; ^2^Dipartimento di Psicologia dello Sviluppo e della Socializzazione, University of PadovaPadova, Italy

**Keywords:** political ideology, authoritarianism, motivated social cognition, dual process models, automatic processing, intergenerational transmission, parental influence

## Abstract

Research on adult populations has widely investigated the deep differences that characterize individuals who embrace either conservative or liberal views of the world. More recently, research has started to investigate these differences at very early stages of life. One major goal is to explore how parental political ideology may influence children's characteristics that are known to be associated to different ideological positions. In the present work, we further investigate the relations between parents' ideology and children cognitive processing strategies within the framework of political ideology as motivated social cognition (Jost et al., 2003) and the dual process model of political ideology (Duckitt et al., 2002). Specifically, epistemic (implicit attitudes toward order vs. chaos), existential (negativity and threat bias), and relational needs (conformity measure) were assessed in pre-school children (*N* = 106; 4–6 years). For each child at least one parent completed both the Social Dominance Orientation (SDO) and the Right Wing Authoritarianism (RWA) measures. Interestingly, results indicated that mothers' and fathers' responses had unique associations with children's socio-cognitive motivations, and different findings emerged in relation to the two facets of parental authoritarianism, namely dominance (i.e., SDO) and submission (i.e., RWA). More specifically, children's existential needs appeared to be more related to mothers' RWA scores, whereas children's epistemic needs appeared to be more related to fathers' SDO. Finally, parents' RWA and SDO scores appeared to have opposite effects on children's relational needs: children's conformity increased at increasing levels of mothers' RWA and decreased at increasing levels of fathers' SDO. Overall, however, results were relatively weak and several links between the responses of parents and their children were not significant, suggesting caution in drawing strong conclusions about the impact of parents' ideology. Limitations and future developments will be discussed.

## Introduction

In the last decade, there has been a renewed interest in the study of political ideology from the psychological perspective (e.g., Jost et al., 2008b). More specifically, research has largely documented how political ideology is correlated with several distinct psychological (e.g., threat sensitivity, Jost et al., 2007), cognitive (e.g., need for cognitive closure, Jost et al., 2007) and neuropsychological (Amodio et al., 2007; Kanai et al., 2011) features. Overall, the key message of these studies is that political ideology actually goes beyond the political field in a strict sense, and it may be reflected in several aspects of everyday life, such as nonverbal behavior in the context of social interaction and characteristics of living and working spaces (Carney et al., 2008). For this reason, it is important and interesting to investigate when and how these differences may start to emerge in very young children and what their likely determinants are. The aim of the present investigation is indeed to examine these differences in young children (4–6 years), in relation to their parents' political attitudes, within the framework of political ideology as motivated social cognition (Jost et al., 2003) and of the dual process model of ideology and prejudice (DPM; Duckitt, 2001).

### Political ideology as motivated social cognition

According to the model of political ideology as motivated social cognition (Jost et al., 2003, 2009; Jost and Amodio, 2012), ideology meets epistemic, existential, and relational needs of certainty, security, and affiliation. In other words, a dispositional or situational heightened motivation to reduce uncertainty, threat or isolation drives the preference for stability over change and the acceptance of inequalities, which are the two core aspects of conservative ideology.

To start with epistemic motives, individual differences pertaining to the management of uncertainty have been shown to predict conservative positions. Indeed, individuals characterized by higher need for structure (Altemeyer, 1998), higher Need for Cognitive Closure (e.g., Kemmelmeier, 1997; Chirumbolo, 2002; Chirumbolo et al., 2004; Van Hiel et al., 2004; Jost et al., 2007; Leone and Chirumbolo, 2008; Hennes et al., 2012), and lower Need for Cognition (e.g., Sargent, 2004; Federico and Schneider, 2007) appear to also embrace more conservative views of the world. Not only dispositional differences, but also situational factors affect ideological positions. For instance, distractions or high cognitive load induce people to adopt more conservative attributional styles (Skitka et al., 2002).

To continue with security needs, conservatives have also been shown to be more sensitive to threat as compared to liberals. As far as existential fear is concerned, a line of research suggests that death anxiety tends to be associated with political conservatism, right-wing authoritarianism and system justifying beliefs (Wilson, 1973; Jost et al., 2003, 2007; Hennes et al., 2012). Experimental studies also showed that manipulations of mortality salience (e.g., Landau et al., 2004; Cohen et al., 2005; Nail et al., 2009) and terrorism salience (Ullrich and Cohrs, 2007) tend to shift people's political opinions and preferences toward conservatism (but see also Kosloff et al., 2010; Lambert et al., 2010 for different results). Besides fear, disgust is also associated to political ideology. Indeed, both correlational and experimental studies (Hodson and Costello, 2007; Inbar et al., 2009, 2012; Terrizzi et al., 2010; Helzer and Pizarro, 2011), indicated that individuals holding conservative opinions are more sensitive to disgust than liberals.

In a related vein, conservatives are more likely than liberals to perceive the world as a dangerous place (Altemeyer, 1998; Duckitt et al., 2002; Mirisola et al., 2014) and to interpret ambiguous facial stimuli as expressing threatening emotions (Vigil, 2010). Conservatives are also more cautious in exploring novel situations and give more weight to negative as compared to positive information in impression formation tasks (Shook and Fazio, 2009; Castelli and Carraro, 2011; Carraro et al., 2014). In addition, conservatives have been shown to display an automatic selective attention to negative stimuli (Carraro et al., 2011; for a review cf. Hibbing et al., 2014).

Finally, relational motives also seem to predict the adoption of conservative ideology (see Jost et al., 2008a). For example, right-wing and authoritarian people have been found to value conformity better than liberals (Feldman, 2003; Cavazza and Mucchi-Faina, 2008) and those endorsing conservative views also have a higher need to achieve a shared view of reality (Hennes et al., 2012). In addition, an experimental study (Jost et al., 2008a) showed that affiliation motives could actually affect political ideology. Indeed, students who had imagined an interaction with their more conservative parent subsequently displayed higher system-justifying beliefs than those who had imagined an interaction with their liberal parent.

Concluding, research involving adult respondents has overall demonstrated that conservatives are characterized by higher epistemic, existential and relational needs as compared to liberals. We thus expected that children of conservative parents would also be more likely to display higher need for certainty, security, and affiliation than children of liberal parents.

### The two sides of authoritarianism

The investigation of the structure of socio-political and socio-cultural attitudes and values (for a review, cf. Duckitt, 2001) typically elicited two relatively orthogonal dimensions broadly corresponding to Right Wing Authoritarianism (RWA; Altemeyer, 1981, 1988) and Social Dominance Orientation (SDO; Sidanius and Pratto, 1993, 1999). Initially conceived as a personality dimension (Adorno et al., 1950; Altemeyer, 1981), RWA is now considered as a collection of generalized attitudes and beliefs (e.g., Duckitt, 2001; Duckitt et al., 2002; Van Hiel et al., 2004), likewise SDO, which was originally designed as such.

According to the DPM (Duckitt et al., 2002; Duckitt and Sibley, 2009), RWA and SDO scales capture two distinct and relatively independent ideological attitude dimensions, stemming from different situational factors, personality traits, and social worldviews. Individuals high on social conformity (vs. autonomy, or openness to experience in Big-Five terms) tend to believe that the social world is dangerous and threatening and thus to pursue the motivational goals of security and social control (vs. personal freedom and autonomy) expressed in high RWA scores. Therefore, RWA is also labeled as authoritarian submission. On the other hand, individuals high on tough-mindedness (vs. tender-mindedness, or agreeableness in Big-Five terms) tend to perceive the world as a ruthlessly competitive jungle and thus to pursue the motivational goals of power, superiority over others and dominance (vs. care, share and help) expressed in high SDO scores. Therefore, SDO is also labeled as authoritarian dominance.

In the present study, we measured both authoritarian submission (i.e., RWA; Altemeyer, 1981, 1988) and authoritarian dominance (i.e., SDO; Sidanius and Pratto, 1993, 1999) in order to achieve a more nuanced assessment of the links between parental ideology and children's socio-cognitive responses.

### Parental influence in the domain of political attitudes

It is generally assumed that political ideology is to a large extent transmitted from parents to children (Adorno et al., 1950; Altemeyer, 1988). A large sample longitudinal set of studies including three generations (Jennings and Niemi, 1968; Jennings et al., 2009) confirmed that, besides the indirect parental influence exerted by placing their children in a given sociopolitical milieu, parents can also have a strong direct influence on children's political learning. This direct influence is likely to be exerted through both socialization and genetic transmission. As for the former process, for instance, Adorno et al. (1950) assumed that parenting styles are both antecedents and consequences of authoritarianism. Indeed, although there is only very weak evidence of an association between parents' ideology and parenting styles (Boshier and Izard, 1972), the latter have been shown to affect children's political ideology (e. g., Miklikowska and Hurme, 2011; Fraley et al., 2012). As for the genetic transmission, several studies (e.g., Eaves and Eysenck, 1974; Tesser, 1993; Olson et al., 2001; Bouchard et al., 2003; Alford et al., 2005; Kandler et al., 2012), comparing monozygotic and dizygotic adult twins, indicate that political ideology can also be genetically determined, with heritability explaining about 50% of the variance in relation to attitudes toward several political issues.

In addition, there are some clues of specific intergenerational transmission for the described two sides of the authoritarianism coin, SDO and RWA. Indeed, young adults' and adolescents' levels of RWA (Altemeyer, 1988; Peterson and Duncan, 1999; Duriez et al., 2008) and SDO (Duriez et al., 2008) have been found to correlate with their parents' responses on the correspondent measures. More importantly, in line with the 90,000 DPM (Duckitt et al., 2002), research indicated that authoritarian submission and dominance are rooted in different socialization experiences. Indeed, Duckitt (2001) has shown that punitive and strict parenting is associated to RWA (through social conformity and dangerous-world beliefs), while neglecting and unaffectionate parenting is associated to SDO (through tough-mindedness and competitive-jungle beliefs).

In a similar way, though from a different perspective, Duriez et al. (2008) found that these distinct dimensions of authoritarianism were specifically predicted by different parental goal promotion efforts that were linked, in turn, to parents' ideology. In particular, conservation (vs. openness to change) goal promotion mediated parent-child correlation in RWA, while extrinsic (vs. intrinsic) goal promotion mediated parent-child correlation in SDO. Focusing on the outcomes of socialization experiences, Weber and Federico (2007) also showed that anxious attachment styles predicted RWA while avoidant attachment predicted SDO, through the correspondent worldviews respectively (but see also Roccato, 2008; Lorito and Falgares, 2013).

It is worth noting that this research on parent-child resemblance in RWA and SDO, such as that on parent-child resemblance in other measures of political ideology (e.g., Jennings and Niemi, 1968; Acock and Bengtson, 1978; Jennings et al., 2009), involved young adults or adolescents as offspring. This is consistent with the general assumption that sociopolitical orientation crystallizes in young adulthood (e.g., Altemeyer, 1998). However, there is reason to predict that those epistemic (e.g., need for closure), existential (e.g., threat sensitivity), and relational (e.g., social conformity) needs that are known to be associated to ideology in adult populations may also be detected among children as a function of the ideological placement of their parents. To date, only two studies tested this hypothesis. In the former (Reifen Tagar et al., 2014), 3–4 year old children's deference to convention and authority was investigated as a function of parents' authoritarian and social conformity values. As predicted, Reifen Tagar et al. (2014) found higher sensitivity to adults' conventionality and status, when deciding whether to trust them in a labeling objects game, among children of parents who were high (vs. low) in authoritarianism and social conformity values. In another study, Dennis et al. (2015) examined 5–7 year old children's neurocognitive responses to conflict as a function of parental ideology and emotional context. Their results showed larger N2 amplitudes (indicating enhanced recruitment of cognitive resources to detect and resolve conflict) in threatening conditions among children of liberal parents as compared to children whose parents embrace more conservative or moderate political views. These results show that psychosocial and neural differences associated with parents' political views can be detected at a very young age, long before the development of sophisticated political beliefs. These fascinating recent lines of research (Reifen Tagar et al., 2014; Dennis et al., 2015), suggesting that a psychological predisposition to embrace certain political views is already present at pre-school age, prompted us to further investigate the differential effects of parents' authoritarianism dimensions on their very young children.

### The present study

Drawing on the previously described research findings, we here tested the general hypotheses that children of conservative parents would display greater needs for certainty, security, and affiliation as compared to children of liberal parents. In operational terms, given that conservative adults, compared with liberal ones, are more attentive to threatening stimuli (e.g., Carraro et al., 2011), exhibit a stronger implicit preference for order vs. chaos (Jost et al., 2008b) and have more positive attitudes toward social conformity (e.g., Cavazza and Mucchi-Faina, 2008), we expected that children of conservative (vs. liberal) parents would score higher on measures of negativity/threat bias (assessed as proxies of existential needs), implicit attitudes toward order vs. chaos (as a proxy of epistemic needs), and social conformity to peers (as a proxy of relational needs). Since it is not clear whether conservatives are biased toward negative stimuli in general or only in relation to threatening stimuli (Lilienfeld and Latzman, 2014), we included measures that enabled us to assess attentional biases elicited by both threatening stimuli and negative stimuli that are not expected to trigger any threat.

Moreover, besides these general hypotheses, we also explored how the distinct dimensions of parental authoritarianism (i.e., SDO and RWA) might be differentially associated to the various dependent measures administered to the children. To the best of our knowledge, past research is not very informative about the differential effect of the socio-cognitive motives underlying ideology Jost et al., 2003 on authoritarian submission and dominance (but see Van Hiel et al., 2004 for an exception). These constructs are often addressed by separate literatures, which have not yet been fully integrated, but specific hypotheses can be put forward. First, we expected that parental RWA, as rooted in a view of the world as a dangerous place, would predict children's sensitivity to threat and could predict their sensitivity to negativity. Second, we expected that parental SDO, as based on a perception of the world as a competitive jungle, might lead to a higher need for order in children, foreshadowing a preference for hierarchy as a form of regularity and arrangement. In addition, following Van Hiel et al. (2004) who showed that adults' need for simple structure predicted not only their SDO but also their RWA score, we hypothesized that parental RWA would also be associated with children's need for order. Finally, as for the relational needs of children, we expected that whereas parental authoritarian submission, valuing obedience and conventionalism, should increase children's tendency to conform, authoritarian dominance, characterized by a need of prevailing and being superior, might reduce children's social conformity to peers.

Finally, in the present study we separately investigated the impact of mothers' and fathers' ideology on children's responses. Indeed, having an independent assessment of ideology from both parents is crucial on order to estimate the role of each parental figure while controlling for the eventual influence of the other parent. The scarcity of previous studies prevents us from formulating straightforward hypotheses, although some research findings suggest that mothers might be in general more influential than fathers (Acock and Bengtson, 1978; Castelli et al., 2009).

## Materials and methods

### Participants and procedure

This study was carried out in accordance with the recommendations of the ethical committee for psychological research at the University of Padova. We recruited participant families in 4 kindergartens in a medium sized town in Northern Italy. One-hundred and six children^1^ (50 males) aged 4–6 years (*M* = 5.18, *SD* = 0.56) were individually interviewed in a quiet room by a researcher after having obtained written permission from the parents in accordance with the Declaration of Helsinki. Children were asked to perform a series of tasks (presented as games) on a computer laptop. In order to simplify the task, we used a keyboard with three different colored labels on the 3 response keys (blue for the left key, yellow for the central key and red for the right key). At least one parent for each child filled in a questionnaire at home and returned it in a closed envelope. Overall, we obtained responses from 85 fathers and 102 mothers, aged 24–62 years (*M* = 41.17, *SD* = 5.52), for a total of 83 complete triads. As for the educational level of the involved parents, 40.2% of them declared a senior high-school leaving accreditation and 41.8% had a university level degree.

### Parents' measures

Parents completed the Italian version (Roccato et al., 2009) of the Right Wing Authoritarianism scale by Funke (2005; 12 items) and an Italian short version (Di Stefano and Roccato, 2005; 8 items) of the Social Dominance Orientation Scale (Pratto et al., 1994). Responses were provided on 5-point Likert scales ranging from “completely disagree” to “completely agree.” We computed average scores for each measure for both mothers (RWA α = 0.63; SDO α = 0.81) and fathers (RWA α = 0.64; SDO α = 0.87).

As measures of authoritarianism could be inflated by social desirability bias (Taylor, 1961), we followed other authors (e.g., Duriez and Van Hiel, 2002; Ekehammar et al., 2004) in administering the Short Marlowe-Crowne Social Desirability Scale (Reynolds, 1982; 13 item). Participants answered on 5-point Likert scales ranging from “completely false” to “completely true.” An average score was computed for each parent (mothers α = 0.62; fathers α = 0.74).

### Children's measures

#### Implicit attitudes toward order vs. chaos

**Figure 1:**
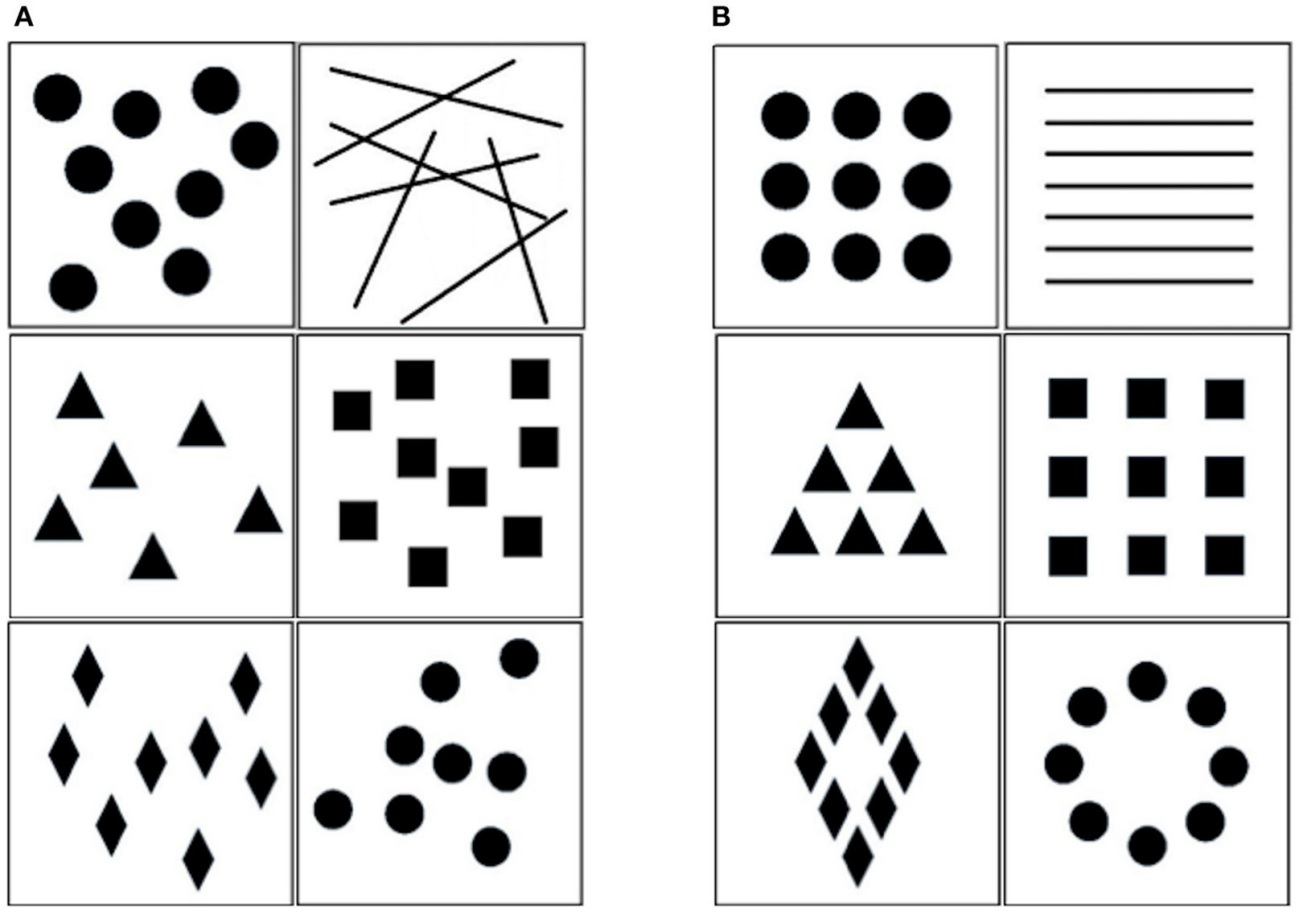
**Target stimuli used in the IAT. (A)** Disordered geometrical figures. **(B)** Ordered geometrical figures.

Children first completed an *Implicit Association Test* (IAT; Greenwald et al., 1998) involving a sequence of five trial blocks (three practice and two critical blocks). Each trial block included instructions about the category discriminations and key assignments. In the first block (practice block), participants categorized attribute stimuli as happy vs. sad emoticons. In the following block (practice block), they had to categorize ordered vs. disordered sets of geometrical figures (6 stimuli for each category; see Figure 1). The third block (critical block) required a combined categorization task, and all stimuli were presented (emoticons and geometrical figures). A specific response key had to be pressed if either positive emoticons (i.e., happy) or ordered figures appeared on the screen, whereas another response key had to be pressed if negative emoticons (i.e., sad or angry) or disordered figures were displayed (i.e., compatible block for participants with a preference for order vs. chaos). In the fourth block (practice block), the response keys for pictures of ordered and disordered figures were reversed as compared to the second trial block. The fifth and final block (critical block) was again a combined categorization task, but this time positive emoticons and disordered figures, on the one hand, and negative emoticons and ordered figures, on the other, shared the same response key (i.e., non-compatible block for participants with a preference for order vs. chaos). The order of the third and fifth critical block, as well as the order of the second and the fourth block, were counterbalanced across participants, so that half of them performed the compatible block prior to the non-compatible, as reported above, whereas the other half performed the non-compatible prior to the compatible block. The practice blocks included 12 trials and the test blocks included 40 trials each. A similar IAT has already been used with adult participants (Jost et al., 2008a).

During this task, the experimenter constantly monitored the children while they performed the initial practice phases of the IAT. When it was evident that participants had not well understood the task (e.g., they always pressed the same response key), the experimenter gave the instructions a second time and invited the children to go through the practice phases again. Seventeen children repeated the at least one of the first two practice blocks and then completed the task smoothly. Only one child continued to have problems in performing the task and therefore the critical blocks were not administered. Moreover, three children interrupted the task because they reported to be tired and their data were thus excluded from the analyses.

Before computing the individual attitude score, we dropped one participant who made more than 30% of errors in the critical blocks. We computed an IAT score accordingly to the algorithm proposed by Greenwald et al. (2003) in such a way that positive values indicate a preference for order rather than chaos. The reliability of the IAT was good and comparable to that observed in the case of adult populations (α = 0.81).

It has to be remarked that the IAT has been successfully used in several previous studies with preschool-aged children in relation to a wide-range of attitude objects (e.g., racial attitudes, gender attitudes, attitudes toward flowers and insects, attitudes toward minimal groups; e.g., Baron and Banaji, 2006; Cvencek et al., 2011; Dunham et al., 2011; see also Williams and Steele, 2016) suggesting that the IAT can actually measure significant aspects of children's implicit social cognition.

#### Social conformity

**Figure 2:**
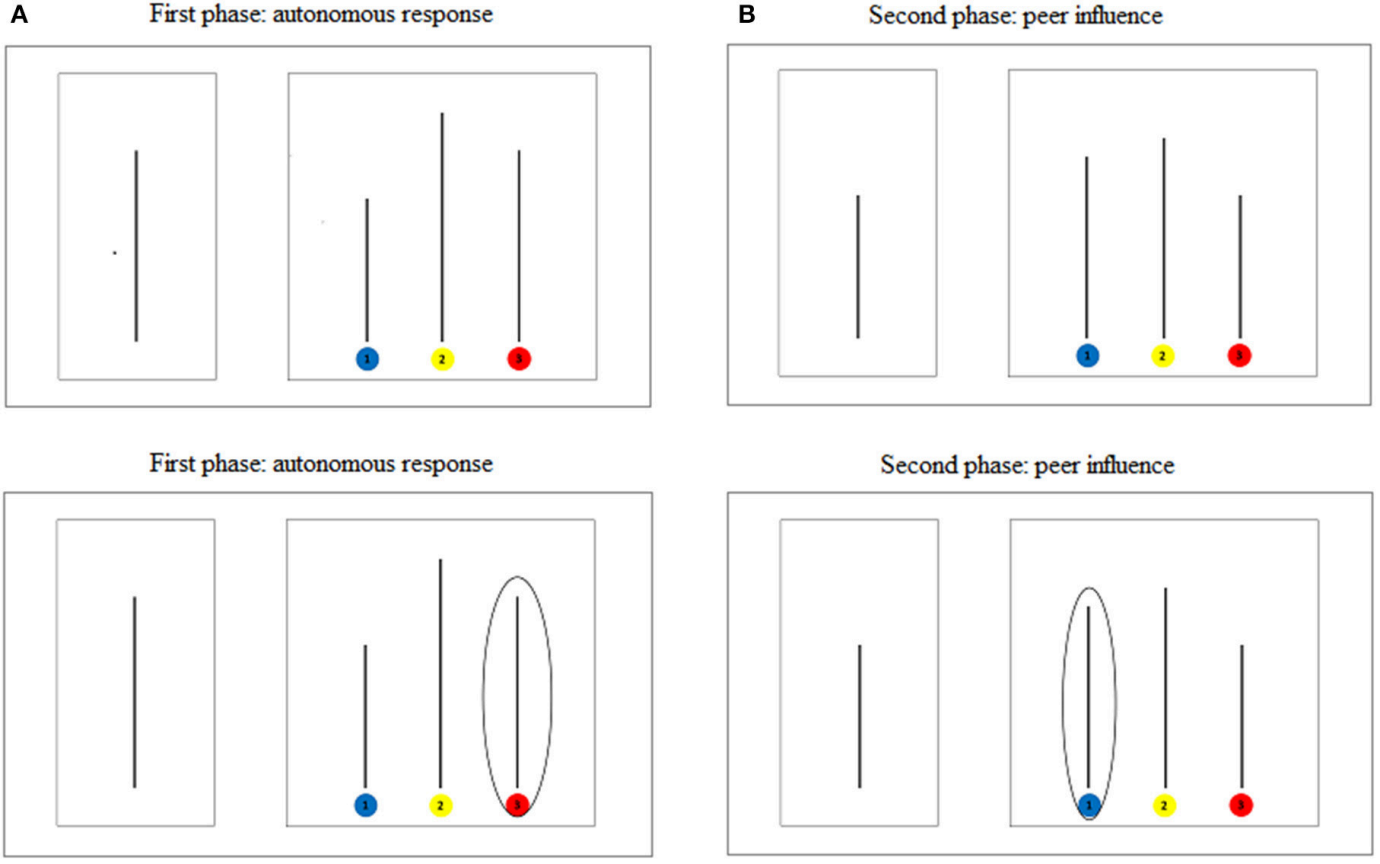
**Example of stimuli used in neutral (A)** and experimental **(B)** trials of the social conformity task.

Social conformity was assessed through an adapted version of a conformity task (Asch, 1956; see also Walker and Andrade, 1996). Overall, participants were presented with 8 trials (2 practice trials, 3 neutral trials and 3 experimental trials; see Figure 2). In the first phase of each trial (i.e., autonomous response), children were presented with one (standard) line appearing on the left half of the computer screen, and were asked to indicate which of three (comparison) lines appearing on the right was of equal length. The responses were given by pressing one of the 3 keys (on the computer keyboard) that were painted with the same colors identifying the 3 comparison lines. The task was very easy and the correct response was never ambiguous. In the second phase of each trial (i.e., peer influence), children were informed about “the responses most of other children of their age had given in the same trial.” The computer provided the information by circling the line that had been allegedly selected by most children and the experimenter only explained the meaning of that circle. These peer judgments were correct in the first 2 (practice) trials and then alternatively correct (3 neutral trials) and incorrect (3 experimental trials) in the remaining 6 trials. After being informed about their peers' bogus responses, participant children were asked to select again the comparison line which they perceived as having the same length as the standard line, by pressing the correspondent colored key (pressured response). When children changed their first correct response and complied with peers by adopting their incorrect response, this was considered as our key index of conformity. We thus computed a social conformity score (ranging from 0 to 3) by summing the number of conformist pressured responses, but only for those participants (*N* = 70)^2^ who always gave correct autonomous responses in the first phase of each trial (autonomous response).

#### Negativity/threat bias

Finally, after completing an unrelated measure about new foods acceptance for different purposes, children performed two Dot-probe tasks (MacLeod et al., 1986), one in which positive and negative but not threatening images were presented, and one in which positive and threatening images were employed. A pilot study allowed us to select the stimuli (the pictures are available upon request to the first author). Thirty-five children (68.6% girls) aged 4–7 (*M* = 5.18; *SD* = 1.03) were asked to evaluate a series of 35 images, each depicting a well-known Walt Disney character. For each character, children reported whether they knew him/her (yes/no), what his/her name was or in which cartoon they had seen him/her, whether s/he was good or bad, pleasant or unpleasant, whether they liked him/her (yes/no), and whether they thought s/he is scary for children their age (yes/no). We then selected as positive stimuli the characters known by most children and judged by most of them as good, pleasant, liked and not scaring. The characters known by most children and judged as bad, unpleasant, disliked and not scaring were selected as negative stimuli; threatening stimuli were considered those negative stimuli (i.e., bad, unpleasant, and disliked) that were additionally perceived as scaring by most children. Overall, 6 stimuli for each category were selected.

In the Dot-probe tasks, participants were initially presented with a fixation cross for 300 ms and then two characters belonging to two different categories (either positive vs. negative but not threatening, or positive vs. negative and threatening) were simultaneously presented; one stimulus appeared on the left side of the screen, whereas the other appeared on the right side. The relative position of the positive and negative stimulus was randomized across trials. After 800 ms the pictures of the two characters disappeared and a gray dot was displayed on one side of the screen where one of the two pictures had just been shown. Participants were instructed to “catch the little mouse” (appearing as a dot because it was very small) by pressing either the left or the right key, according to the side where the “mouse” was presented. After participants had provided their response there was a 500 ms interval before the following trial started. The relative order of the 2 Dot-probe tasks, involving either negative but not threatening stimuli or negative and threatening stimuli, in addition to positive stimuli, was counterbalanced across participants. The first Dot-probe task that was administered was preceded by an 8-trials practice block, while each test block included 40 trials.

The rationale was that responses would be faster when the dot was displayed on the side where participants' attention was already allocated (compared to the other side). If negative pictures draw spatial attention to their location (relative to the location of positive pictures), we should expect faster reaction times to targets displayed on the same side of negative rather than positive stimuli. Before computing the scores, we dropped 5 participants who made more than 10% of errors (*N* = 95). Following Elam et al. (2010) we also discarded trials with reaction times shorter than 150 ms or longer than 2000 ms. We then computed, for each participant, both a “negativity bias” and a “threat bias” score using the following equation: (right probe following left negative/threatening image − right probe following right negative/threatening image) + (left probe following right negative/threatening image−left probe following left negative/threatening image)/2 (see MacLeod et al., 1986). Positive values indicated that participant's attention was more attracted by negative/threatening stimuli than by positive stimuli.

Children were asked to complete the described tasks in the following order: the IAT aimed at assessing implicit attitudes toward order and chaos, the conformity task and the two Dot-Probe tasks measuring negativity and threat bias.

## Results

Descriptive statistics for each variable are reported in Table 1, whereas correlations among measures are reported in Table 2. Interestingly, mothers' and fathers' scores were significantly correlated. The coefficients suggest that parents share some similarity in their political ideology but also that the overlap is only partial, thus highlighting the importance of analyzing the influence of each parent on children's responses while controlling for the potential influence of the other parent.

**Table 1:** **Descriptive statistics**.

	***N***	**Min**	**Max**	***M***	***SD***
IAT order vs. chaos	100	−0.76	2.52	0.82	0.71
Threat bias	95	−157.18	356.35	10.51	82.84
Negativity bias	95	−245.78	137.99	−13.95	7.73
Social conformity	70	0.00	3.00	1.00	1.10
Mother RWA	102	1.33	3.67	2.71	0.53
Mother SDO	102	1.00	5.00	1.97	0.70
Father RWA	85	1.42	4.08	2.81	0.56
Father SDO	86	1.00	4.50	1.96	0.78
Mother social desirability	102	2.17	4.69	3.40	0.49
Father social desirability	86	1.46	4.69	3.40	0.58

**Table 2:** **Correlations among measures, for all participants**.

	**2**	**3**	**4**	**5**	**6**	**7**	**8**
1. IAT order vs. chaos	−0.072	−0.095	−0.072	−0.060	−0.030	−0.059	0.300^**^
2. Threat bias		0.042	0.256^*^	0.206^*^	−0.063	0.031	0.046
3. Negativity bias			−0.238	0.024	−0.069	0.030	0.083
4. Social conformity				0.175	0.042	−0.093	−0.284^*^
5. Mother RWA^a^					0.442^***^	0.427^***^	0.282^**^
6. Mother SDO^a^						0.308^***^	0.527^***^
7. Father RWA^a^							0.278^***^
8. Father SDO^a^							

a *Non-standardized residuals of each measure regressed on social desirability score*.

* p < 0.05;

** p < 0.01;

*** *p < 0.001*.

Preliminary analyses showed that there was no difference in children's responses as a function of school, class, age, number of brothers/sisters and birth order. Only for the IAT measure, assessing the attitudes toward order vs. chaos, the main effects of gender, *t*_(98)_ = 2.44, *p* = 0.016, and blocks order, *t*_(98)_ = 5.93, *p* < 0.001, were statistically significant: Male respondents displayed more positive implicit attitudes toward order (*M* = 0.99, *SD* = 0.64) as compared to female respondents (*M* = 0.65, *SD* = 0.73), and IAT scores were generally higher when the compatible block was presented first (*M* = 1.16, *SD* = 0.61) than when it was presented after the non-compatible block (*M* = 0.43; *SD* = 0.61). For this reason, we controlled for the effects of gender and blocks order only in the main analyses involving the IAT measure.

In addition, in all the analyses, we controlled for parents' tendency to provide socially desirable responses; to this end, we regressed parental RWA and SDO on their Social desirability scores, saved the non-standardized residuals and then used these adjusted variables in the subsequent analyses in which such variables were entered as predictors of children's responses. Results of these linear regression analyses are reported in Table 3.

**Table 3:** **Results of linear regressions on children's measures predicted by parents' measures**.

	**IAT order vs. chaos**			**Threat bias**			**Negativity bias**			**Social conformity**		
	**β**	***t***	***p***	**β**	***t***	***p***	**β**	***t***	***p***	**β**	***t***	***p***
(Constant)		4.81	0.000		1.01	0.316		−2.40	0.019		7.89	0.000
Child's gender	−0.26^**^	−2.81	0.006									
Blocks order	0.51^***^	5.38	0.000									
Mother SDO^a^	−0.12	−1.07	0.290	−0.14	−0.94	0.351	−0.19	−1.27	0.210	0.11	0.70	0.489
Mother RWA^a^	−0.05	−0.43	0.666	0.29^*^	2.03	0.046	−0.03	−0.19	0.847	0.37^*^	2.33	0.024
Father SDO^a^	0.30^**^	2.77	0.007	0.08	0.57	0.573	0.21	1.42	0.161	−0.41^**^	−2.85	0.006
Father RWA^a^	−0.16	−1.55	0.126	−0.09	−0.64	0.524	0.03	0.20	0.845	−0.17	−1.18	0.245
*R*^2^	0.44^***^			0.06			0.04			0.23^*^		

a *Non-standardized residuals of each measure regressed on parent's social desirability score*.

* p < 0.05;

** p < 0.01;

*** *p < 0.001*.

### Implicit attitudes toward order vs. chaos

Results of linear regression analyses showed that fathers' SDO scores were predictive of children's IAT scores, so that children displayed a stronger preference for order as their fathers' level of authoritarian dominance increased. No effect was found in relation to mothers' SDO, as well as in relation to the RWA scores of both mothers and fathers.

### Social conformity

As for the social conformity task, the RWA score of mothers (but not that of fathers) significantly and positively predicted children's responses, while the SDO score of fathers (but not that of mothers) negatively predicted them. In other words, children were more conformist to allegedly erroneous peers as their mothers' level of authoritarian submission increased and their fathers' level of authoritarian dominance decreased.

### Negativity/threat bias

Finally, the RWA score of the mothers was a positive and significant predictor of children's threat bias, whereas the RWA of the fathers was unrelated to children's responses in the task (see Table 3). Interestingly, no effect emerged in relation to SDO confirming, as predicted, that authoritarian submission, as captured by responses to the RWA scale, is a stronger predictor, as compared to SDO, of the tendency to carefully monitor for the presence of threatening stimuli in the environment. Neither SDO nor RWA scores were related to responses in the Dot-probe task in which negative but not threatening stimuli were presented. This further suggests that mothers' RWA is likely to specifically influence children's security needs, as indexed by their tendency to carefully monitor for stimuli that are not only negative but also able to elicit threat.

## Discussion

The present study explored the general hypothesis that 4–6 years old children might differ in their socio-cognitive motivations as a function of parental ideology, differentiating between two facets of ideology, namely authoritarian submission (i.e., RWA) and authoritarian dominance (i.e., SDO). Results were only partially in line with the main prediction regarding the relevance of parents in the transmission of ideology-related characteristics among children. In particular, interesting distinct results were obtained in relation to right-wing authoritarianism and social dominance orientation of mothers and fathers, although with several caveats that will be later discussed.

Children were administered three tasks aimed at assessing their socio-cognitive motivations. More specifically, automatic attention to threatening stimuli was used as a proxy for assessing children's existential need for security, implicit preference for order vs. chaos was a proxy of epistemic need for certainty, and behavior in a conformity to peer task was intended to tap relational need for affiliation.

To start with needs for security and certainty, only mothers' RWA scores (but not mothers' SDO scores and fathers' SDO and RWA scores) appeared to be significant predictors of children's level of threat bias whereas only fathers' SDO (but not fathers' RWA scores and mothers' SDO and RWA scores) was a predictor of the implicit attitudes toward order vs. chaos. This pattern of results might be explained on the basis of the different underpinnings of RWA and SDO. When mothers perceive the world as a dangerous place, as indexed by high scores on RWA, this might increase their children's threat bias and anxiety when exposed to frightening stimuli (e.g., de Rosnay et al., 2006). In the case of fathers, their view of the world as a competitive jungle, that would benefit from a more defined structure and hierarchy, seems to be associated to children's stronger need for order and regularity. This is partially inconsistent with the results by Van Hiel et al. (2004), showing that individuals' need for simple structure predicted not only their SDO but also, and even more strongly, their RWA score. It should be stressed that previous research has mainly focused on university students and adult respondents, whereas still limited data is available in relation to preschool-aged children.

The fact that different patterns emerged in relation to the RWA and SDO scores assessed from the two parents was not specifically predicted. Nonetheless, the obtained findings seem to be consistent with the idea that each parent exerts a distinctive influence in different domains (O'Bryan et al., 2004). More specifically, more emotionally-related responses (i.e., threat bias) were influenced by the female parent who is stereotypically associated to the emotional sphere. In contrast, the need for order and control over the environment (i.e., implicit attitudes toward order vs. chaos) was influenced by the male parent who is stereotypically considered as more agentic in the structuring of the social and physical environment. This is admittedly a *post-hoc* tentative explanation and future research is needed to address both the consistency and meaning of the reported finding.

In addition, the link between mothers' RWA and children's responses in the Dot-probe tasks was restricted to situations in which threatening stimuli were presented. Indeed, when negative but not threatening pictures were displayed no effect emerged, suggesting that mothers' authoritarian submission does not play a key role in relation to an overall evaluative dimension (i.e., when stimuli are either positive or negative) but only when the stimuli are able to trigger strong emotional responses, as in the case of threatening stimuli.

Children were also asked to complete a social conformity task, as a proxy for assessing relational needs. Interestingly, the two aforementioned sides of the authoritarianism coin, as measured by the RWA and SDO scales, had opposite relations with children's social conformity. Indeed, conformity increased at increasing levels of authoritarianism submission (i.e., RWA) and decreased at increasing levels of dominance (i.e., SDO). Moreover, mothers and fathers appeared again to exert specific influences in line with gender stereotypes, as discussed above. In particular, mothers' RWA appeared to be a positive predictor of children's conformity, whereas fathers' SDO was a negative predictor of the same measure. In other words, mothers who value obedience and conventionalism, as they view the world as a dangerous place, seem to boost children's relational needs of affiliation and thus their tendency to follow their peers, even when they provide incorrect responses. Conversely, fathers characterized by a need of prevailing and being superior, as they view the world as a competitive place, seem to dampen their children needs of affiliation and thus their tendency to conform to incorrect peers; in contrast, they seem to promote a desire to confirm the correct response that provides order and stability in the perception of the world. It is worth noting that each parent's influence seems to be exerted through the gender stereotypical dimension of authoritarianism: submission (which is expected from women) for mothers and dominance (which is expected from men) for fathers.

These results on social conformity are in line but also extend previous evidence by Reifen Tagar et al. (2014). Indeed, they found that children of parents high in authoritarian and social conformity values, were more likely to trust and follow both a conventional and an ambiguously conventional adult in labeling some objects. In other words, children of more conservative parents were more conformists to adults in general and to conventional adults in particular. In the current study, we assessed children's tendency to conform to unknown peers and not to adults who adhered to convention (Reifen Tagar et al., 2014). This allows us to tap two different crucial aspects of conformity behavior in relation to the two different faces of authoritarianism, submission and dominance.

To summarize, the present study showed that mothers with stronger authoritarian-submissive tendencies had children displaying higher vigilance to threatening stimuli and higher level of conformity toward incorrect peers, suggesting that they may have high existential and relational needs for security and affiliation; in contrast, fathers with stronger authoritarian-dominant tendencies had children displaying more positive implicit attitudes toward order vs. chaos and lower level of conformity toward incorrect peers, suggesting that they may have high epistemic needs for certainty and a stronger tendency to reaffirm their autonomous view of the world. Concluding, current results suggest once again (see Reifen Tagar et al., 2014; Dennis et al., 2015) that parents may start shaping the “*political ideology*” of their children from a very early stage of development, well before political issues become a topic of explicit discussions between parents and their children.

### Limitations and future directions

As previously remarked, this study only represents an initial exploration of the influence of parents' authoritarianism dimensions on children's socio-cognitive responses and thus several limitations must be acknowledged. First, one might appropriately observe that RWA scale is tridimensional and thus is not a “pure” measure of submission (Duckitt and Bizumic, 2013). However, it has to be noted that we used a brief version of the RWA scale, with only 12 items (Funke, 2005; Roccato et al., 2009). This scale has been found to have a three-dimensional factorial structure (defined by authoritarian submission, authoritarian aggression, and conventionalism), but the authors of the Italian validation (Roccato et al., 2009) highlighted the poor reliability of the three substantive factors and the very strong correlations among them, thus warning against mechanically considering the brief version of the scale as three-dimensional. Therefore, we also decided to calculate a single score for the RWA scale, observing that the reliability of the overall scale was relatively low, but still acceptable (i.e., α = 0.63 for mothers and α = 0.64 for fathers), whereas the reliability of the subscales was very low.

In addition, it has to be acknowledged that despite the significant results reported and discussed above, in the current study several effects were non-significant. Therefore, even the single significant effects that have been presented here should be placed in the context of an overall fuzzy picture. For this reason, future research recruiting a wider sample is necessary in order to further investigate and better understand the relations between parents' SDO and RWA and their children socio-cognitive motives. Moreover, the current mixed results may also indicate that the relations between the studied variables are very complex and somehow relatively weak and, as a consequence, unstable. It is likely that other variables do play an important role and thus future studies may include some moderators while analyzing these relations. Our position is that the mixed findings presented in the present paper, although suggestive, should be taken with extreme caution being in no way conclusive about an actual link between parents' ideology and children's socio-cognitive responses. Nevertheless, both the significant and non-significant results obtained in the current study may represent important additional elements for a comprehensive meta-analytic approach to the issue that might hopefully provide more conclusive responses.

Finally, it has to be noted that this is a cross-sectional correlational study, thus causal relations cannot be determined. Although mutual influence cannot be excluded (Miklikowska, 2016), we can reasonably assume that parental ideological beliefs are more likely to affect children's responses, rather than the reverse, at least in the case of very young respondents, such as the preschoolers involved in our study. However, longitudinal research is necessary in order to provide more straightforward results not only about the casual relations but also about the development stability of these embryonic differences. Some studies have already shown that some features detected in children (e.g., early temperament) may be correlated with political ideology in adulthood (e.g., Block and Block, 2006; Fraley et al., 2012) suggesting that children's behaviors can actually be predictive of later more sophisticated political attitudes.

Notwithstanding these limitations, the present work adds to the recent and so far limited literature suggesting the existence, at a very young age, of ideology-based differences in children of conservative and liberal parents, also providing some hints about the possible different and specific influences of the two parents and of the two different aspects of authoritarianism.

## Author contributions

Conceived and designed the experiments: MG and LCar. Performed the experiment: MG. Analyzed the data: MG, LCar, and LCas. Wrote the first draft of the paper: MG, LCar, and LCas.

## Funding

This research was financially supported by the Italian Ministry of Education, University and Research (MIUR; Futuro in Ricerca, 2012 Grant RBFR128CR6).

### Conflict of interest statement

The authors declare that the research was conducted in the absence of any commercial or financial relationships that could be construed as a potential conflict of interest.

## Footnotes

^1^The exact number of participants changes across tasks because not all the children completed all the tasks. Moreover, some participants were discarded in some tasks on the basis of the percentage of incorrect responses.

^2^Ninety-seven children were administered the task. Nine children did not complete the whole task because either reported to be tired or verbally indicated that they had not understood the instructions even after repeated explanations. Twenty-seven children made at least one mistake in the autonomous response and were thus excluded from the analyses (22 children made 1 mistake and 5 made 2 mistakes).
